# 

**Published:** 1899-09

**Authors:** 


### EDITORIAL.

### National Dental Association.

The National Dental Association will hold its next annual meeting at Old Point Comfort, Va., commencing June 26, 1900. The election of officers resulted as follows:

President, B. Holly Smith, Baltimore, Md.; vice-president from the East, John J. Hart, New York City; vice-president from the West, Truman W. Brophy, Chicago; vice-president from the South, M. F. Finley, Washington; corresponding secretary, Emma Eames Chase, St. Louis, Mo.; recording secretary George H. Cushing, Burbank, Cal.; treasurer, Henry W. Morgan, Nashville, Tenn.; executive committee, H. A. Smith, J. D. Patterson (to succeed themselves), S. F. Waters; executive council, H. J. Burkhart, Thomas Lillebrown, J. Y. Crawford, J. S. Cassidy, W. E. Griswold.

### The 13th International Medical Congress.

The 13th International Medical Congress will meet in Paris, August 2 to 9, 1900. This Congress will have a section of Stomatology, to which will be admitted French and foreign doctors practicing this branch.

The officers of the organizing committee are: President, Dr. Pietkiewecz; Vice-Presidents, Drs. Cruet and Gaillard; Secretary-General, Dr. Ferrier.

All communications or questions concerning this section should be addressed to Secretary-General, Dr. Ferrier, 39, r. Boissy-d'Anglas, Paris.

We hope, in the next issue of the REGISTER, to give a more definite account of the program of this meeting. It is presumed that there will be quite a large number of dentists of this country who will arrange to attend the meeting, as there will be many attractions in Paris at this time. There will be extensive travel next year, and transportation will doubtless be very greatly reduced from the ordinary rates. We hope in the near future to know more of the precise rates that will be provided.

